# Electrochemical ammonia oxidation using nickel copper hydroxide with H_2_ recovery at high current density and selectivity

**DOI:** 10.1039/d5gc06877k

**Published:** 2026-05-01

**Authors:** D. D. van Noordenne, P. J. Jungbacker, A. Urakawa, F. M. Mulder

**Affiliations:** a Chemical Engineering, Faculty of Applied Sciences, Delft University of Technology 2629 HZ Delft The Netherlands f.m.mulder@tudelft.nl

## Abstract

Electrochemical conversion of ammonia has received increasing attention due to the potential applications for fertiliser production and wastewater treatment. This work demonstrates the application of a homogeneously copper-doped nickel hydroxide, prepared through an easily scalable precipitation method, as an electrochemical oxidation catalyst. A cation exchange membrane divides the cell and prevents re-reduction of the oxidation product. During chronopotentiometry Ni_0.8_Cu_0.2_(OH)_2_ was able to perform ammonia oxidation, with limited oxygen evolution, from 2.5 mA cm^−2^ up to 400 mA cm^−2^. The faradaic efficiency for nitrite formation increased with the applied current density. At a high initial ammonia concentration of 1 M, Ni_0.8_Cu_0.2_(OH)_2_ converted 77% of the ammonia in less than 3.5 hours, applying a high current density of 400 mA cm^−2^. This resulted in a faradaic efficiency of 96% total, which is 91% NO_2_^−^ and 5% NO_3_^−^, which would be impossible in an undivided cell. Therefore, this work demonstrates the potential for efficient and selective ammonia oxidation towards nitrite under industrially relevant current density and conditions.

## Abstract 2

Electrochemical ammonia oxidation to nitrite using a homogeneous nickel copper hydroxide catalyst with H_2_ recovery at high current density.

## Graphical abstract

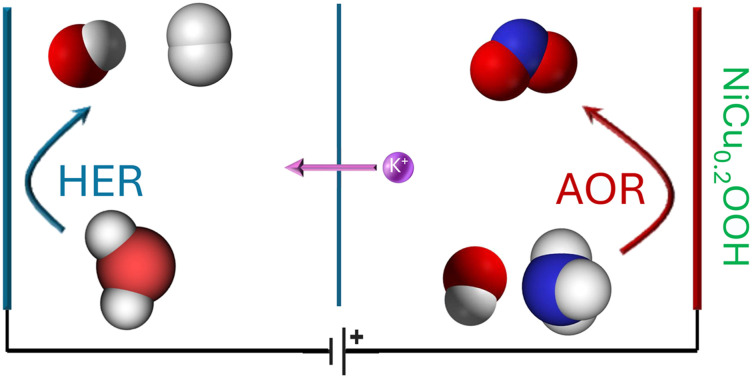

**Green foundation**

1. The electrochemical oxidation of ammonia at high selectivity to nitrite and at industrially relevant current densities is presented, with coproduction of green hydrogen for recovery of energy.

2. Nitrogen oxides and salts are used intensively as fertilisers and chemical feedstocks and provide about four times higher value per nitrogen unit than ammonia. When produced using green electrons, these materials can be obtained in a green manner.

3. It is shown that high ammonia concentrations in 1 M KOH electrolyte can be utilised, going beyond concentrations encountered in wastewater treatment. A cation exchange membrane cell design with Ni_0.8_Cu_0.2_OOH as the active catalyst effectively protects the oxidation products from undesirable reduction.

## Introduction

Humanity relies on the production of synthetic fertilizers to sustain the growing population.^1^ In 2021, 187 million tons of ammonia was produced, with 147 million tons used for synthetic fertilizer manufacture. While ammonia is mainly used directly in fertilizer production, a significant amount is converted to nitric acid. This conversion resulted in 70 million tons of nitric acid in 2021.^2^ Most of the nitric acid is combined with ammonia to produce synthetic ammonium nitrate fertilizers.

As renewable energy is increasingly implemented, increasing interest exists in producing ammonia and nitric acid electrochemically. Nonetheless, direct electrochemical ammonia production from nitrogen gas and water has yet to reach significant and reproducible synthesis rates, compared to the optimized Haber–Bosch process.^3–6^ The main challenge is the difficulty of dinitrogen activation, before even being able to optimize the process. However, it would be more feasible to replace the subsequent ammonia oxidation to nitrite or nitrate using electrochemistry as one then starts from more electrochemically active ammonia. Currently, large-scale conversion is performed in the multistep Ostwald process, starting with oxidation with O_2_ at 10 bar and 800 °C with a Pt/Rh gauze.^7,8^ The formed NO is cooled down and reacted with the remaining oxygen to form NO_2_. This NO_2_ is absorbed with water to form nitric acid. As an alternative in this last step, the application of a hydroxide solution results in nitrite salt rather than the commonly produced nitric acid.^9^ In comparison with these process conditions, electrochemistry could make it feasible to produce nitrite and nitrate with more benign conditions and a catalyst consisting of more Earth-abundant elements. Furthermore, local application of the product, combined with small-scale ammonia, aligns with distributed renewable electricity generation. Local production and application could reduce the transport costs of both electricity and chemical products. Therefore, it is interesting to investigate electrochemical ammonia oxidation.

The electrochemical ammonia oxidation reaction (AOR) has been reported for various applications, such as fuel cells and wastewater treatment.^10–18^ In these fields, noble metals are commonly used to achieve high selectivity and conversion to N_2_ (eqn (1)). Recent research has shifted focus toward converting ammonia to nitrite (eqn (2)) and nitrate (eqn (3)).^19–22^ The oxidation potentials are presented in SI Note S1. The applied conditions, such as concentration, faradaic efficiency (FE) and current density, vary significantly in the literature due to the aim to recover ammonia and nitrate from various wastewater streams (Table S1). Nickel is of significant interest as a precious metal-free catalyst that can achieve high efficiency and selectivity. Furthermore, nickel is corrosion resistant due to the stability of its solid oxidised species under alkaline and oxidative conditions. The active form is NiOOH, which can either react electrocatalytically (eqn (4)) or result in indirect oxidation with the reformation of Ni(OH)_2_ (eqn (5)) and subsequent reoxidation of Ni(OH)_2_ to NiOOH (eqn (6)).^23^ The competing OER reaction and potential above which it can take place is also given (eqn (7)). At the counter electrode, water reduction produces H_2_ and OH^−^ (eqn (8)).1
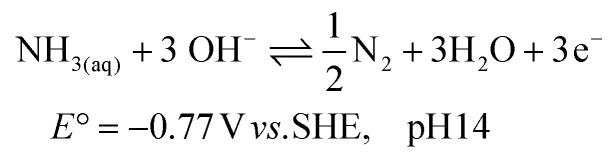
2
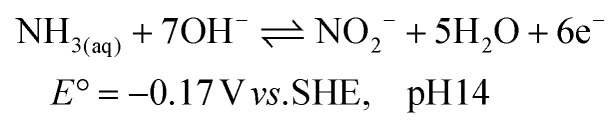
3
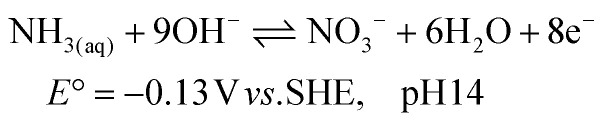

4 NiOOH + NH_3_ → NiOOH + X + *n*H^+^ + *n*e^−^

5 6NiOOH + NH_3_ + H_2_O + OH^−^ → 6Ni(OH)_2_ + NO_2_^−^

6
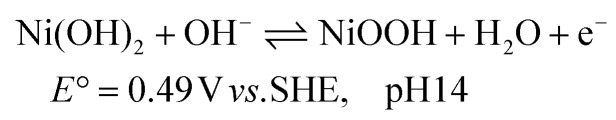
7
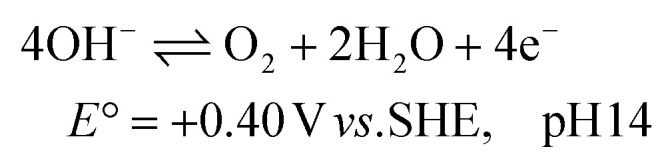
8
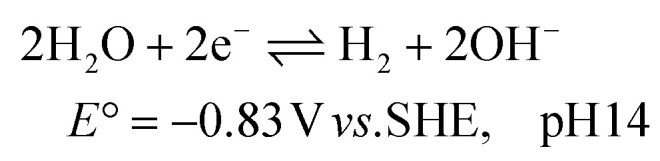


The layered nickel hydroxide structures have been doped with various metals to improve their properties.^19,21,24–26^ The most promising approach is the addition of copper to the nickel hydroxide, resulting in a catalyst that is highly active for ammonia oxidation. For example, Jiang *et al.* reported selectivity up to 99% towards nitrite for wastewater denitrification under relevant conditions of 1 mM NH_3_ and 0.1 M KOH electrolyte.^20^ However, they presented and analysed a nanostructured phase separated catalyst material of Ni(OH)_2_ and Cu(OH)_2_. Possibly at the interface of these materials, there could be an activity enhancing phase compared to the two pure phases.

In our research, we found that it is possible to produce a homogeneous Cu doped Ni_1−*x*_Cu_*x*_(OH)_2_ phase. Compared to the heterogenous mixture, one may expect that now all the material has the influence of Cu doping and this could thus result in improved activity with respect to the pure and inhomogeneously Cu-doped Ni(OH)_2_ catalysts. Here, we report the application of a homogenously copper-doped nickel hydroxide electrode, Ni_0.8_Cu_0.2_(OH)_2_.

**Fig. 1:**
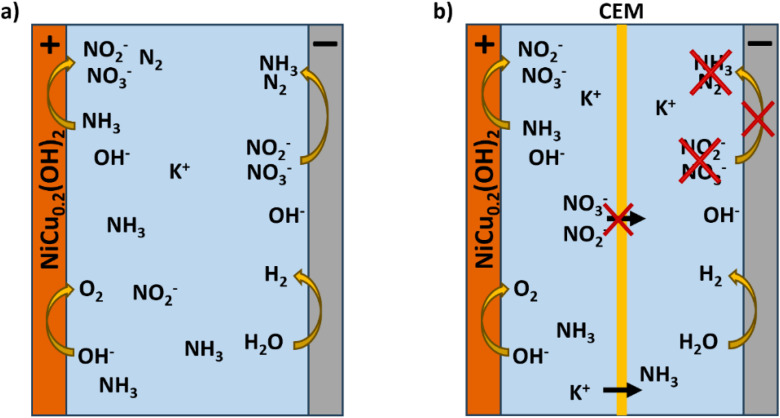
The difference between performing the AOR in an alkaline electrolyte within (a) an undivided cell and (b) a cell divided with a cation exchange membrane. The ionic current can be carried by K^+^, while the product anions NO_2_^−^ are blocked. NH_3_ in the feed can only react at the positive electrode. At alkaline pH, NH_3_(aq) remains.

Besides the catalyst, the experimental cell can significantly influence the results achieved with the AOR, which is commonly performed in a single cell compartment. In such a configuration, the formed nitrite and nitrate anions remain dissolved in the electrolyte and are reducible at the counter electrode to NH_3_ and N_2_ (Fig. 1a). To prevent this, it will be shown that a cation exchange membrane (CEM) should be applied to prevent such anion crossover and re-reduction of the oxidation products (Fig. 1b). Within this study, we shall establish the advantages of applying a divided CEM cell for the AOR compared to an undivided cell and also show the high conversion and current densities that can be achieved with the homogeneous Ni_0.8_Cu_0.2_(OH)_2_ catalyst. The CEM appears crucial to achieve high product selectivity and FE and to determine the FE unambiguously. Furthermore, we show that it becomes possible to work with concentrated ammonia solutions and current densities up to 400 mA cm^−2^.

## Experimental

All chemicals were obtained from Sigma-Aldrich and were used as purchased. All water mentioned is Mili-Q water with a conductivity of <0.1 µS cm^−1^. A 28 wt% NH_3_ concentrated solution was used. A high concentration solution of ammonia in 1 M KOH was prepared by slowly mixing the appropriate KOH solution and 28 wt% NH_3_. The resulting solution was kept in a closed container to minimize ammonia losses.

### Synthetic procedures

To obtain a homogeneously mixed Ni–Cu phase material, a previously reported co-precipitation method was used and modified for the addition of Cu instead of Fe.^27^ An aqueous solution containing NiSO_4_·6H_2_O (0.25 M, 50 mL, 20.6% Ni) with the corresponding amount of CuSO_4_ (0.0625 M, 99%) was prepared to obtain the Ni_0.8_Cu_0.2_(OH)_2_ material. Under vigorous stirring of the KOH (2.0 M, 100 mL) solution, the metal precursor solution was added dropwise. The precipitate was collected by centrifugation (3500 rpm, 10 min) and after washing twice with water and once with ethanol. The residue was dried at 50 °C under vacuum. Ball milling at 200 rpm for 15 min resulted in a powdered material for preparing the electrodes.

### Material characterisation

X-ray diffraction was performed using a Bruker D8 Advance ECO diffractometer equipped with a Cu-K_α_ source (K_α1_ = 1.54060 Å, 40 kV and 25 mA) and a Lynxeye-XE-T position sensitive detector. A fixed divergence slit was used with a Bragg–Brentano geometry. A step size of 0.02° with a measuring time of 0.1 s per step was employed. SEM and EDS were performed on both the powders and electrodes using a SEM-JEOL-6010LA.

### Electrode preparation

Electrodes were prepared by homogenously mixing the material (Ni_1−*x*_Cu_*x*_(OH)_2_) with 50 wt% carbon super P® and 50 wt% graphite through pestling. The binder polyethersulfone (7 wt% in *N*-methylpyrrolidone) was added to obtain a slurry. The nickel foam matrices (0.5 mm thick, 99.9% Ni) were prepared by cutting 20 × 20 mm squares. These squares were cleaned in HCl (4 wt%) and acetone for 5 min each in a sonication bath. After pasting, the slurry was solidified through phase inversion in water.^28,29^ Such an electrode processing method is known to result in highly porous electrodes with a large electrochemical surface area, which is advantageous for relatively high current densities. The electrodes were dried under vacuum at 50 °C.

### Electrochemical experiments

Cyclic voltammetry was performed with a Parstat MC-1000 system with a three-electrode setup. The Tafel slope and chronopotentiometric measurements were carried out using a Maccor 4000 battery cycling system. These were mainly performed in a three-electrode setup. Auxiliary potential channels could be connected to measure cell potential and the potential of the counter electrode in comparison with a second Hg/HgO reference electrode.

Cyclic voltammetry and measurements without membranes were performed in a single compartment cell with a nickel foam counter electrode and an Hg/HgO (KOH) reference electrode. Experiments with a membrane were performed with a 20 mm diameter Nafion 117 membrane in between two 50 mL electrolyte compartments. If ammonia was added, it was added on both sides of the membrane to reduce the effect of initial differences in the ammonia concentration and pH on the measurements, and to demonstrate the limited poisoning effect of ammonia on the counter electrode. Before performing ammonia oxidation during chronopotentiometry, the cell was operated at the same current density without ammonia until a stable potential was achieved. This was to stabilize the electrode in the nickel oxyhydroxide state and convert all potential nitrogen contamination^30^ to nitrogen gas, nitrite, and nitrate to improve accuracy.

Cyclic voltammetry was performed in 1 M KOH in the range of 0–0.63 V *vs.* Hg/HgO at a scan rate of 1 mV s^−1^. This was followed by the addition of 50 mM NH_4_OH and repeated. The fifth cycle was used for analysis. The Tafel slope measurements were performed by initially applying a current to convert all hydroxide in the catalytic material into oxyhydroxide before applying a constant current for 10 min at increasing current densities. A range from 0.25 mA cm^−2^ up to 25 mA cm^−2^ was applied, where the area refers to the geometric surface area.

### Ion chromatography

The concentration of anions in the electrode were determined using an ion chromatograph (IC). The system consisted of a Thermo Scientific™ Dionex™ Integrion™ HPIC™ system equipped with a conductivity detector and an AS18-Fast anion column. In accordance with Thermo Scientific application note 72481, AutoNeutralization™ was applied to improve accuracy by removing the KOH background without dilution. A gradient in the KOH concentration of the eluent was applied to obtain completely separate nitrite and nitrate peaks for maximum accuracy.

## Results and discussion

**Fig. 2:**
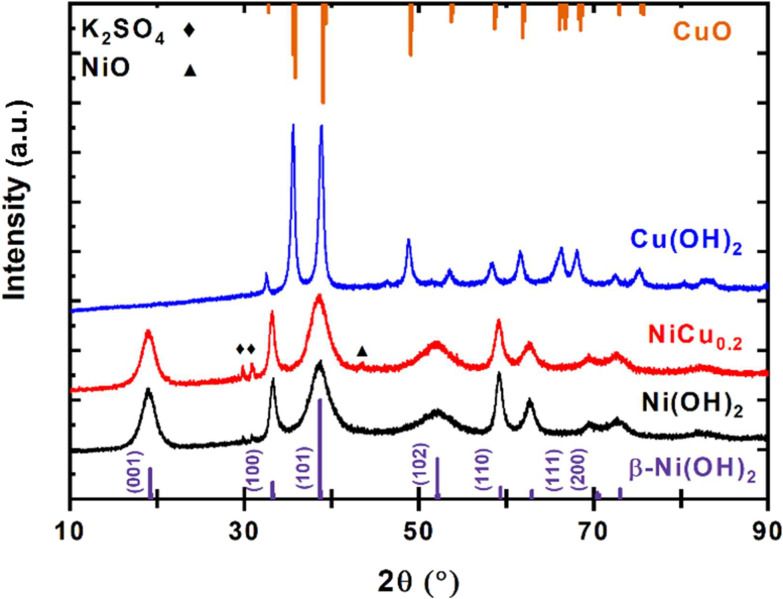
XRD pattern of NiCu_0.2_(OH)_2_ in comparison with those of β-Ni(OH)_2_ and Cu(OH)_2_ recorded through the same synthesis method. At the bottom, a β-Ni(OH)_2_ (COD 1011134) reference pattern, and at the top, a CuO (COD 1011148) reference pattern, are shown. The NiCu_0.2_(OH)_2_ pattern closely resembles that of β-Ni(OH)_2_.

X-ray diffraction (XRD) was used to confirm the formation of Ni_0.8_Cu_0.2_(OH)_2_, denoted as NiCu_0.2_, synthesized through a precipitation method under alkaline conditions. The same synthesis method was applied with the individual nickel and copper salt, which clearly formed two distinguishable materials (Fig. 2). The nickel precipitated as the β-Ni(OH)_2_ phase, consistent with the reference pattern. The copper salt formed CuO, as Cu(OH)_2_ can lose water during drying. The XRD pattern of NiCu_0.2_ indicates the formation of β-Ni(OH)_2_ with a potentially minimal peak shift, indicating the successful doping of the copper. The absence of a separated phase suggests that the Cu is homogenously incorporated.

**Fig. 3:**
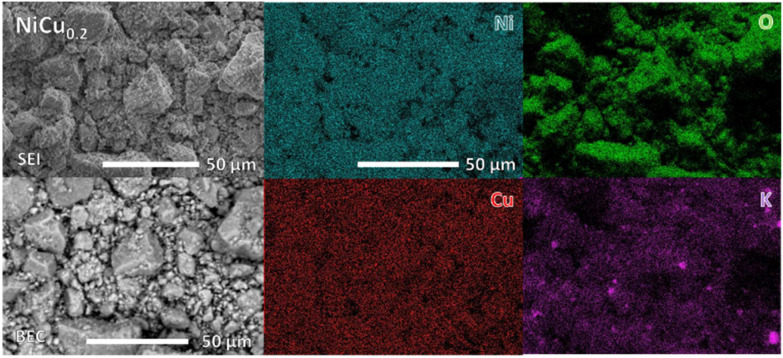
SEM and EDS images of NiCu_0.2_ with separate images, indicating the presence of Ni, Cu, O and K. The inhomogeneous distribution of K correlates with sulphur and originates from the separated K_2_SO_4_ salt from the precipitation synthesis method. Both secondary electron imaging (SEI) and backscatter electron imaging (BEC) modes of SEM are applied to show topography and composition, respectively.

Homogeneous incorporation of the copper was further confirmed with SEM and EDS (Fig. 3). The NiCu_0.2_ particles consist of crystallites but the presence of a background indicates also an amorphous material in between. EDS analysis shows a homogeneous distribution of Ni and Cu, suggesting that copper is homogenously incorporated into the Ni(OH)_2_. Furthermore, a Ni : Cu ratio of 0.83 : 0.17 throughout the powder was determined. This is close to the theoretical ratio. Potassium and sulphate are present as a crystalline K_2_SO_4_ contaminant from the precipitation method. This salt was further washed out during the electrode preparation (Fig. S1). Overall, these results demonstrate copper homogenously incorporated to form NiCu_0.2_.

**Fig. 4:**
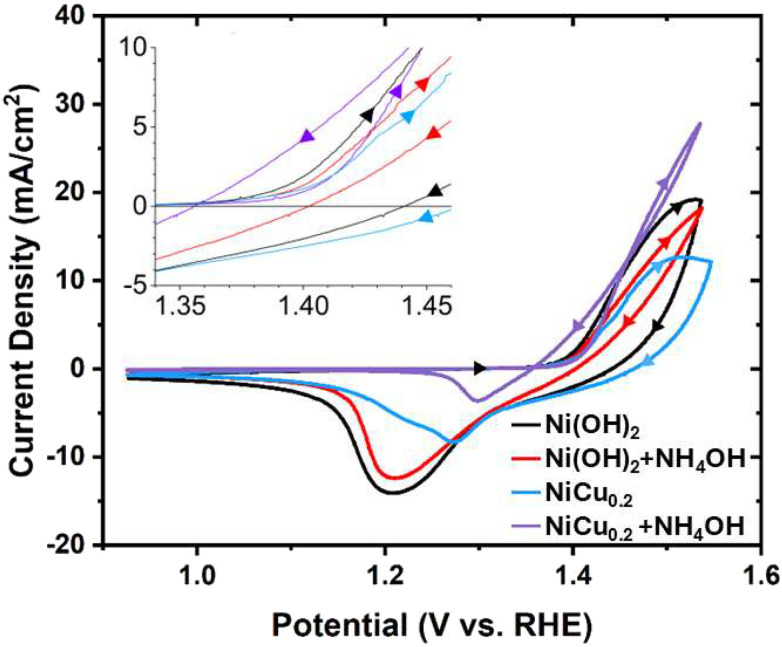
Cyclic voltammetry of Ni(OH)_2_ and NiCu_0.2_ in 1 M KOH with and without 50 mM NH_4_OH. A scan rate of 1 mV s^−1^ was used. The current densities refer to the geometric surface area.

**Fig. 5:**
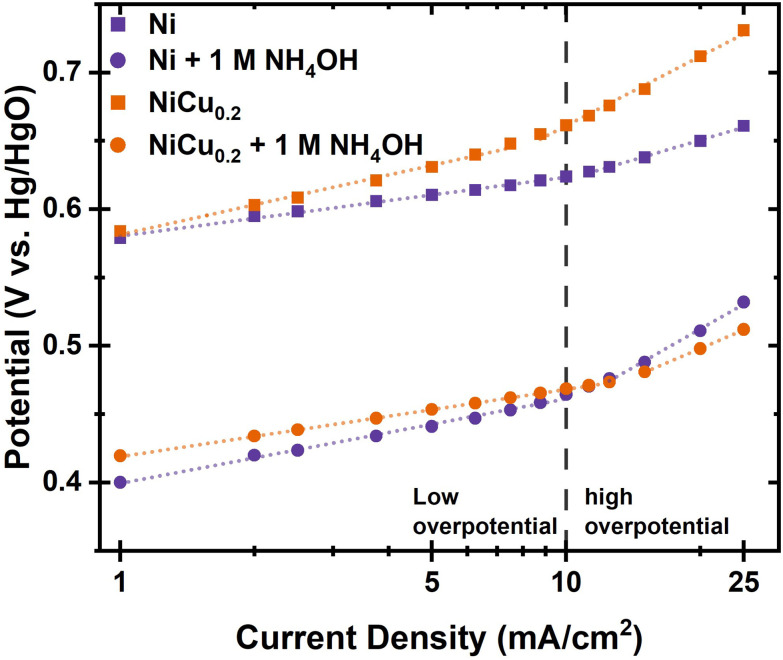
The Tafel plot of Ni(OH)_2_ and NiCu_0.2_ for the OER and the AOR in 1 M KOH with and without 1 M dissolved NH_3_ based on a stepwise increase of current density to reduce error caused by gas bubble formation. The dashed line illustrates the divide between the low and high overpotential ranges.

Cyclic voltammetry was used to investigate the catalyst and its AOR activity (Fig. 4). The Ni(OH)_2_ reference exhibits two redox peaks. The anodic peak is the overlapping Ni^2+^/Ni^3+^ oxidation peak and the OER activity, with an onset potential of 1.39 V *vs.* RHE. The reduction peak originates from the reduction of both γ-NiOOH to α-Ni(OH)_2_ and β-NiOOH to β-Ni(OH)_2_, with the γ-NiOOH reduction occurring at a more positive potential.^31^ In general, γ-NiOOH is associated with higher attainable Ni^3+^ to Ni^4+^ valences, which are charge compensated by the anion and water incorporation in the layered structure. The incorporation of Cu results in a limited change in onset potential of the oxidation peak, but the current density is significantly reduced. Also in the Tafel plots in Fig. 5, it can be observed that the NiCu_0.2_ has lower OER activity at the same applied potential. This lower OER activity is beneficial for suppressing the competing OER and can therefore increase the AOR FE. The cathodic peak shape is different from that of pure Ni(OH)_2_, which seems to be related to more γ-NiCu_0.2_OOH reduction at a more positive reduction potential, compared to less β-NiCu_0.2_OOH reduction. This suggests that significant phase conversion to γ-NiCu_0.2_OOH occurs during oxidation.

The addition of ammonia to the electrolyte resulted in a 10 mV reduction of onset potential for oxidation with β-Ni(OH)_2_. However, the overall peak current density decreased, suggesting that Ni(OH)_2_ has a limited AOR activity. The smaller reduction peak implies that AOR occurs, as less current is available for NiOOH formation. In contrast to Ni(OH)_2_, NiCu_0.2_ has an increased oxidation current density with a significant peak shape change in the presence of ammonia. Initially, the cathodic scan has a similar current density to the anodic scan, before surpassing it as it drops to zero at 1.35 V *vs.* RHE. This enhanced activity originates from the AOR on the oxyhydroxide formed during oxidation, and continues with an indirect AOR below the onset potential of the anodic scan, reforming oxyhydroxide back to hydroxide (eqn (5)). The smaller NiCu_0.02_OOH reduction peak with the presence of ammonia confirms this mechanism. These results indicate that the AOR is limited by the formation of NiOOH from Ni(OH)_2_. The onset potential of the AOR is therefore equal to the potential of NiOOH formation.^20^ The higher AOR activity will then be an intrinsic property of NiCu_0.2_, which can be related to Cu with its valence limited to 2+, affecting the Ni^2+^ : Ni^3+^ : Ni^4+^ ratio and the formation of NiCu_0.2_(OH)_2_ and β- and γ-NiCu_0.2_OOH or a mixed ‘interstratified’ phase.^20,24,32–36^ The remaining cathodic peak is also at the more positive potential side, associated rather with γ-NiCu_0.2_OOH reduction to α-NiCu_0.2_(OH)_2_ than with β-NiCu_0.2_OOH. This may indicate that especially the β-NiCu_0.2_OOH is active in the (indirect) AOR as the β-phase is apparently already reduced following eqn (5) before the cathodic sweep reaches its reduction potential in the absence of NH_3_. Elucidation of the reaction mechanism is challenging and may require *in operando* XRD and EXAFS characterisation methods during the AOR to provide insight into the phase behaviour and state of Ni and Cu,^18^ while *in operando* near ambient pressure XPS could potentially be used to observe relevant N species.^37^

Tafel plots were measured to further investigate the difference in the reaction kinetics between Ni(OH)_2_ and NiCu_0.2_. They were measured with an initial anodic current to convert all hydroxide material to oxyhydroxide, because NiOOH is the AOR active material. In the Tafel plot, a low and high overpotential range is observed (Fig. 5). At low current densities, the Tafel slopes are 43 and 72 mV dec^−1^ for Ni(OH)_2_ and NiCu_0.2_, respectively. The difference is more significant in the high overpotential range, with 96 and 169 mV dec^−1^, respectively. These higher Tafel slopes indicate a lower OER activity for NiCu_0.2_, which should limit OER activity during ammonia oxidation, increasing the FE.

The addition of ammonia results in significant changes in both the Tafel slope and potential. At 10 mA cm^−2^, we found a reduction of potential from 0.624 to 0.465 V and from 0.662 to 0.469 V *vs.* Hg/HgO for Ni(OH)_2_ and NiCu_0.2_, respectively. For Ni(OH)_2_, this coincides with a Tafel slope increase to 62 mV dec^−1^. In the high overpotential range, the Tafel slope changes to 186 mV dec^−1^. The significant increase in slopes indicates sluggish AOR activity with intermediate coverage limiting the OER. In comparison, the Tafel slopes of NiCu_0.2_ reduce to 49 and 140 mV dec^−1^ for the low and high current density ranges, respectively. This explains the higher AOR activity measured with cyclic voltammetry. With this reduction in the Tafel slope, it can be expected that at high current densities, NiCu_0.2_ will be able to maintain high AOR selectivity. This is a result of OER activity being limited by the higher onset potential and Tafel slope. Therefore, we will focus on NiCu_0.2_ to evaluate the effect of experimental conditions and cell layout on the ammonia oxidation.

### Electrolyte and pH dependence of the ammonia oxidation reaction

High AOR activity has been exhibited by NiCu_0.2_. Nonetheless, the supporting electrolyte and ammonia concentration can significantly influence the AOR selectivity and FE. Therefore, understanding the effect of electrolyte is crucial to achieve high FE and selectivity under industrial relevant conditions. The influence of the electrolyte was investigated in a single compartment cell (Fig. 1a) to establish the impact of the electrolyte composition and undivided cell. Each experiment started with achieving a stable potential without the presence of ammonia to form the active NiOOH material, before initiating the experiment with the addition of dissolved ammonia. This method removes apparent FE losses that are solely related to Ni(OH)_2_ oxidation to NiOOH, representing a continuous industrial ammonia electrooxidation process. The nitrite and nitrate production was monitored while the latter concentration was negligible and is therefore omitted for clarity, unless relevant for discussion.

**Fig. 6:**
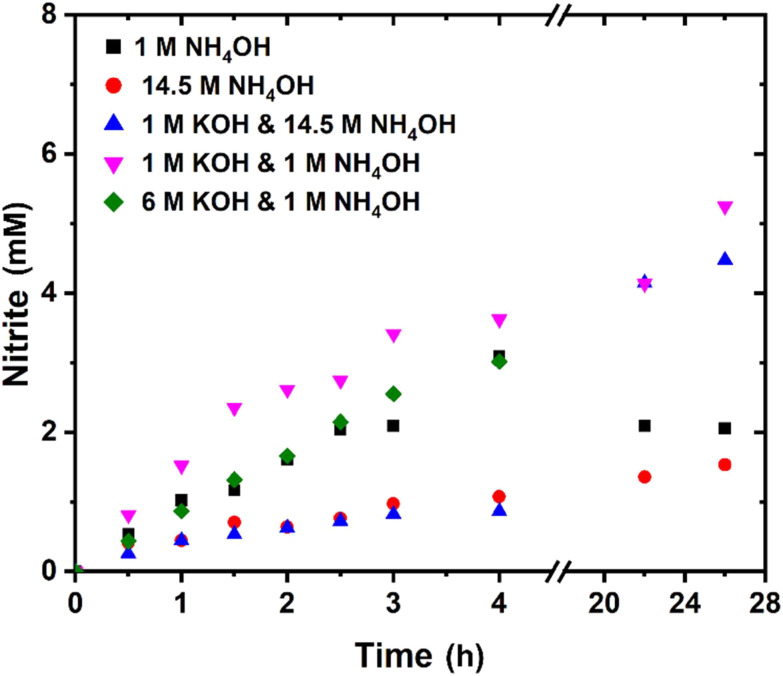
Bulk electrochemical oxidation of ammonia under various conditions at 5 mA cm^−2^ with NiCu_0.2_ as the catalyst. Only the nitrite concentration is shown as the nitrate concentration was negligible unless otherwise mentioned.

The application of 1 M NH_4_OH as the electrolyte resulted in a FE of 34% for the conversion of ammonia to nitrite in the initial 4 hours, with consistent nitrite formation (Fig. 6). However, after 22 hours, the nitrite concentration decreased. This agrees with our earlier statement regarding unobstructed access to the counter electrode resulting in reduction of nitrite, achieving an equilibrium concentration. In comparison, concentrated ammonia (28 wt%, 14.5 M) reduced the nitrite formation rate and a FE of 10% for the initial 4 hours was determined. After 22 hours, only a slight increase in nitrite concentration was observed, implying that the additional ammonia does not promote the reaction. Furthermore, a pH effect was observed. Medvedev *et al.* reported that Ni(OH)_2_ switches to producing nitrate below pH 12.^38^ This is near the pH of the electrolyte with values of 11.6 and 12.2 for 1 M and 14.5 M NH_4_OH, respectively. The pH is further lowered with conversion of the basic ammonia to strong nitrous and nitric acids. This is confirmed by an increase in nitrate concentration to 0.16 and 0.29 mM after 22 hours for 1 M and 14.5 M ammonia, respectively.

**Fig. 7:**
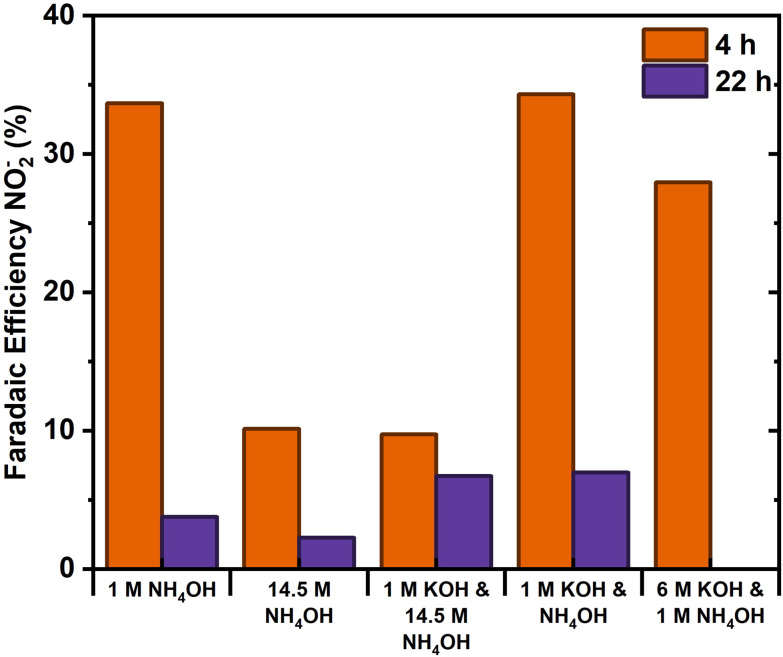
Summary of the faradaic efficiency towards nitrite for the initial 4 hours and the total 22 hours of the abovementioned experiment within a single compartment cell with NiCu_0.2_ at 5 mA cm^−2^.

The results indicate that the addition of an alkaline supporting electrolyte, KOH, is required to stabilize the pH. The addition of 1 M KOH to the 14.5 M NH_4_OH electrolyte resulted in no notable difference within the initial 4 hours. Despite this, after 22 hours, a higher nitrite concentration was established. In comparison, 1 M KOH and 1 M NH_4_OH combined had a significantly higher initial production rate, without noticeable end concentration difference. The initial rate would mainly depend on the direct catalytic effect, suggesting that ammonia or reaction intermediates block active sites with 14.5 M NH_4_OH limiting the rate. The addition of 1 M KOH to 1 M NH_4_OH promoted the AOR, suggesting that the higher pH is not detrimental to the AOR, rather an optimal ammonia concentration between 1 and 14.5 M exists. The similar higher end concentration originates from the OH^−^ ion conductivity, reducing the transport number of nitrite and the correlated nitrite mobility and its (detrimental, nitrite loss making) reduction at the counter electrode. The KOH concentration was further increased to 6 M KOH with 1 M NH_4_OH to investigate if this can enhance nitrite production. However, the nitrite formation rate decreased, likely due to the reduced NH_3_ solubility and increased vapor pressure with higher KOH concentration.^39^ As a result, less NH_3_ is available for oxidation within the electrolyte. The production rate was still linear for 4 hours, confirming that the FE loss does not originate from NH_3_ loss over time. Therefore, we chose to continue with 1 M KOH and 1 M NH_4_OH for the following experiments, as it should result in the highest FE and stability (Fig. 7).

### Cation membrane integration for the increased yield and product separation

1 M KOH limits the experimental runtime due to the change in pH. Therefore, the experiment was repeated with 6 M KOH to determine if prolonged conversion could be achievable. Furthermore, the cell was reduced from 125 to 50 mL for each compartment to improve the performance. This reduction decreased the electrode gap, measuring time and ammonia vapour losses. The vapour loss was further reduced by limiting air contact through further sealing. The experiments were terminated when the initial OER potential, established before ammonia addition, was reached. This resulted in an insignificant ammonia concentration remaining, while preventing electrode damage from the reduced pH.

**Fig. 8:**
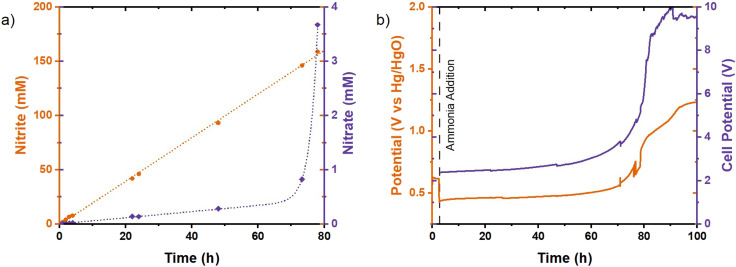
Chronopotentiometry of the NiCu_0.2_ electrode performing the AOR in 1 M NH_3_ in 1 M KOH at 10 mA cm^−2^ in a glass H-cell divided by a Nafion 117 cation exchange membrane. (a) Concentration of products during chronopotentiometry and (b) the measured potentials. The left graph stops at 80 hours, as the results of the last 20 hours were not representative.

An H-cell divided by a Nafion 117 CEM wass chosen as the setup for preventing reduction of nitrite and nitrate at the counter electrode, as this has very limited nitrite and nitrate anion transport and high cation transport. In this case, the K^+^ transport is dominant as it has a high concentration at 1 M KOH and protons are essentially absent. Ammonia was added to both sides of the membrane to mitigate ammonia crossover. The initial results achieved with this setup in 1 M KOH with 1 M NH_3_ electrolyte at 10 mA cm^−2^ were promising with a constant production of nitrite for 70 hours (Fig. 8a). This is a significantly longer runtime with constant production, resulting in a yield of 150 mM nitrite, demonstrating the detrimental effect of product reduction at the cathode in the absence of the membrane. After these initial 70 hours, the nitrate concentration increased exponentially due to the depletion of OH^−^ and reduction of the pH to below 9. This coincided with an increase in potential due to the reduction of conductivity from the lower electrolyte concentration (Fig. 8b). The change in the pH and depletion of OH^−^ is expected under alkaline conditions, because the CEM transfers K^+^ through the membrane, while simultaneously OH^−^ is consumed within the anolyte. NH_4_^+^ could also be transported as a cation; however, at the applied pH, its presence is suppressed in favour of neutral dissolved NH_3_. The pH dependence of nitrite oxidation to nitrate was confirmed by performing an experiment in 1 M KOH with 1 M KNO_2_ (Fig. S4). Only 33 mM nitrate was formed after 6 hours. When the measured oxidation potential had significantly increased, indicating the lowering of pH, almost complete conversion to nitrate was obtained. Therefore, maintaining a stable alkaline pH is crucial to achieve high nitrite selectivity.

**Fig. 9:**
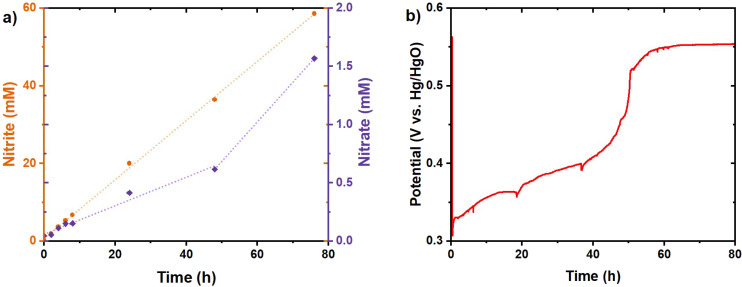
Chronopotentiometry of NiCu_0.2_ in 6 M KOH with 1 M NH_3_ at 2.5 mA cm^−2^. (a) Measured nitrite and nitrate concentrations with the dashed lines showing linear trends. (b) The measured potential during the chronopotentiometry.

**Fig. 10:**
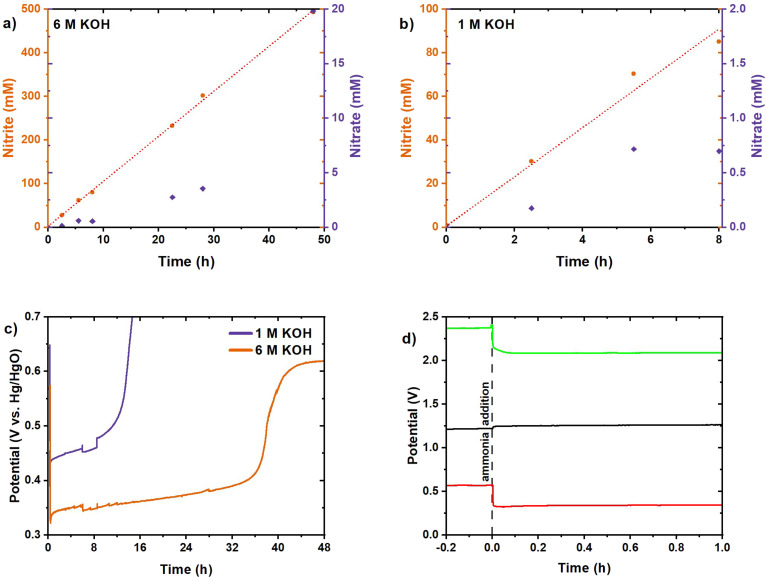
Product concentration for chronopotentiometry with NiCu_0.2_ at 25 mA cm^−2^ in the cation membrane-divided cell: (a) 1 M KOH with 1 M NH_3_ and (b) 6 M KOH with 1 M NH_3_. (c) Potential measured during the chronopotentiometry. (d) Absolute potentials measured during the chronopotentiometric measurement for the first hour in 6 M KOH with 1 M NH_3_. Ammonia addition is illustrated with a dashed line. The working electrode (red) and counter electrode (black) were measured against their own reference electrode. The cell potential is marked in green.

The initial results with 6 M KOH in the electrolyte were obtained with a total operation time of 76 hours with minimal nitrate formation (Fig. 9). A lower FE of 52% at 2.5 mA cm^−2^ towards nitrite was obtained, in comparison with 97% for the previous measurement with 1 M KOH at 10 mA cm^−2^. The limited activity in 6 M KOH could originate from the limited formation rate of NiCu_0.2_OOH due to the lower potential at the applied current density. The reaction was initiated with a potential of 0.33 V *vs.* Hg/HgO. This is just above the NiCu_0.2_(OH)_2_/NiCu_0.2_OOH equilibrium potential for NiCu_0.2_(OH)_2_ at a low state of charge, indicating that a NiCu_0.2_OOH surface could be formed.^27,40,41^ Also when comparing the experiment with a pure Ni(OH)_2_ electrode (Fig. S5), the-Cu doped sample directly shows a higher potential but rather a lower rate as the AOR seems to be potential dependent. In combination with the CV scans in Fig. 4, we conclude that the current and resulting potential are high at the onset of AOR activity, while the increased AOR activity requires higher potentials. In addition, Fig. 5 shows the potential at the same low current density of the NiCu_0.2_ sample to be higher than that for pure Ni, which would bring it closer to that for the OER. Thus, increasing the current density and the correlated potential should increase the NiCu_0.2_OOH formation rate and the subsequent AOR to nitrite, and move into a current and potential territory where the NiCu_0.2_ in the presence of ammonia requires a lower potential for the same current. Therefore, to promote the AOR, the current density was increased to 25 mA cm^−2^ (Fig. 10) and 1 M KOH was compared with 6 M KOH again. The 1 M KOH electrolyte had a limited runtime of 14 hours with a higher potential in comparison with 6 M KOH (Fig. 10b). Furthermore, the potential with 6 M KOH electrolyte was significantly lower than expected based on the pH difference (Fig. 10c). This is expected to originate from the AOR and will result in improved energy efficiency. In the initial 8 hours, the nitrite FE was 87% for 1 M KOH, slightly higher compared to 82% for 6 M KOH. However, the difference in FE is less significant than the potential reduction. The addition of ammonia resulted in a potential drop of 0.20 V with 1 M KOH, compared to 0.24 V with 6 M KOH. This effect was further demonstrated by the cell potential. The addition of ammonia reduced the potential to 2.67 V from 2.76 V with 1 M KOH, whereas a 12% reduction from 2.24 V to 2.08 V was recorded with 6 M KOH. These potentials are higher than the theoretical potential due to activation overpotentials, and the electrode gap and its resulting significant internal resistance. Furthermore, the prolonged stable activity with 6 M KOH resulted in 49% conversion after 48 hours with 78 and 4% FE towards nitrite and nitrate, respectively. These promising results suggested a further increase of the current density as that could result in even higher FE as we approach an industrially relevant current density.

**Fig. 11:**
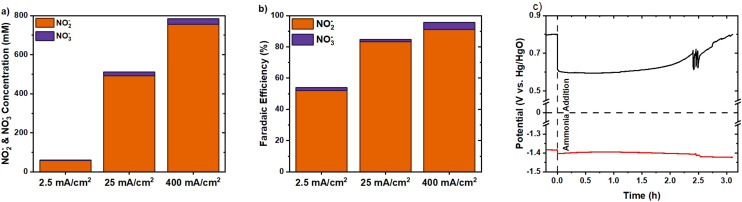
AOR in a cation membrane-divided cell. (a) Nitrite and nitrate concentrations for the chronopotentiometric measurement with NiCu_0.2_, at the end measured in 6 M KOH with 1 M NH_3_ at various current densities. (b) Summary of the faradaic efficiency achieved for the chronopotentiometric methods with increasing current density. (c) Measured potential at a current density of 400 mA cm^−2^ for the working electrode (black) and the counter electrode (red) against their own reference electrode in their compartment.

To further prove the feasibility of industrial electrochemical ammonia oxidation, the current density was increased to 400 mA cm^−2^. Within less than 3.5 hours, 769 mM of ammonia was converted to nitrite and nitrate (Fig. 11a). The initial OER potential of 0.80 V *vs.* Hg/HgO reduced to an AOR potential of 0.60 V *vs.* Hg/HgO upon ammonia addition (Fig. 11c). The limited potential decrease of 20 mV from the counter electrode suggests a minimal poisoning effect on hydrogen evolution. A total cell potential of 2.0 V was calculated, ignoring internal resistances. The theoretical equilibrium cell potential should be 1.3 V, the difference between eqn (6) and (8), as (2) only occurs when NiOOH is present. A significant difference originates from the non-optimised counter electrode and the high overpotential. Further cell optimisation outside this study is required to actually reach reduced potentials closer to the equilibrium potential. At the higher applied current density and potential, a nitrite FE of 91% and a total FE of 95% were achieved (Fig. 11b). Meanwhile, the potential exceeded the onset potential of the OER but did not result in significant OER activity. Ammonia and AOR intermediates limit O_2_ formation while enhancing nitrite formation. This occurs either by occupying actives sides with intermediates and ammonia or by reacting with the OER intermediates.^42^ The OER requires the formation of NiOOH, which is rapidly consumed through an indirect AOR when locally present. This agrees with the lower AOR Tafel slope of NiCu_0.2_, indicating higher activity for the AOR than the OER.

### Comparison with previous results

As stated in the Introduction section, a comparison with the literature for AOR catalysts is presented in Table S1. The AOR towards nitrite and nitrate is reported mostly for Cu and Ni or Co based catalysts, while noble metal-based catalysts mostly yield N_2_. The yields of nitrate and nitrite show a maximum FE of above 86% for the applied current density of 2 mA cm^−2^. The maximum current density applied of ∼100 mA cm^−2^ yields about 5% FE conversion to nitrite. The presently observed 400 mA cm^−2^ and FE of 96% therefore advance the AOR significantly towards industrially relevant values.

Direct comparison is difficult because the Ni–Cu based materials are reported to be inhomogeneous mixtures of Ni(OH)_2_ and Cu(OH)_2_, or Cu–Ni metal alloys. The latter will have an oxidised (oxy-)hydroxide surface since the potentials applied are well above their oxidation potentials. Also, note that their AOR potentials are above the OER/ORR *vs.* RHE (eqn (7)), and therefore more positive than the equilibrium potentials of eqn (1)–(3), excluding spontaneous fuel cell operation. The published onset potential changes in a representative example by Xu *et al.* are small in comparison with *in situ*-grown inhomogeneously mixed Ni(OH)_2_- and Cu(OH)_2_-based nanowires with and without ammonia.^24^ This is also the case for the Ni(OH)_2_ and NiCu_0.2_ reported here as the oxyhydroxide formation determines the onset potential. In addition, the Tafel slopes with 55 mM NH_4_^+^ concentration are similar to those observed with 1 M NH_3_. However, in ref. 24, the current density saturated above a concentration of 150 mM, which means lower currents in the Tafel experiment at higher concentrations. The reported oxidation product at their attainable 9 mA cm^−2^ after 24 h is about 19% FE nitrate, while now with the homogeneously prepared NiCu_0.2_, we report below constant current densities up to 400 mA cm^−2^ and high FE conversion to nitrite. Also, pure Ni(OH)_2_ is reported in membrane-separated cells to reach promising AOR activities up to ∼30 mA cm^−2^ with 0.2 M NH_4_OH, but with still significant N_2_ production (20–90% FE) next to nitrite and nitrate yields that depend on the pH. The OER will make up the missing current amounts at the applied potentials.

### Electrode stability

**Fig. 12:**
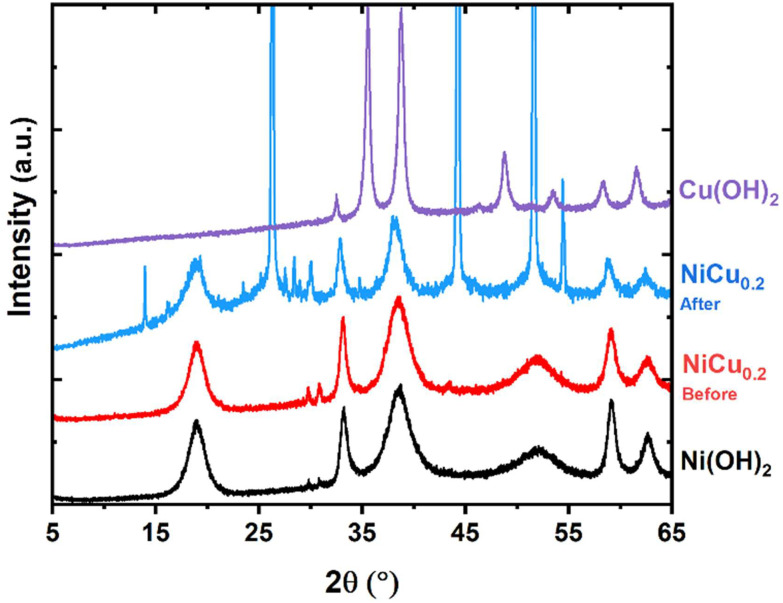
XRD pattern of NiCu_0.2_ used for the experiment with 6 M KOH and 1 M NH_3_ at 400 mA cm^−2^ for close to 3.5 hours. Ni(OH)_2_ and Cu(OH)_2_ powders were added as references for related peaks. The four biggest sharp peaks originate from graphitic carbon and nickel present in the electrode. NiCu_0.2_ powder was added for comparison of the phase and potential peak changes.

Long-term stability of the electrode material is fundamental to ensure cost-effective industrial application. The XRD pattern of the used electrode is comparable to that of the original powder, with the β-Ni(OH)_2_ phase as the main contributor (Fig. 12). Additional peaks correspond to the nickel foam, graphitic carbon, KOH·2H_2_O and KHCO_3_. Furthermore, no separated copper oxide or peak shift was detected, indicating a limited change within the crystalline material.

**Fig. 13:**
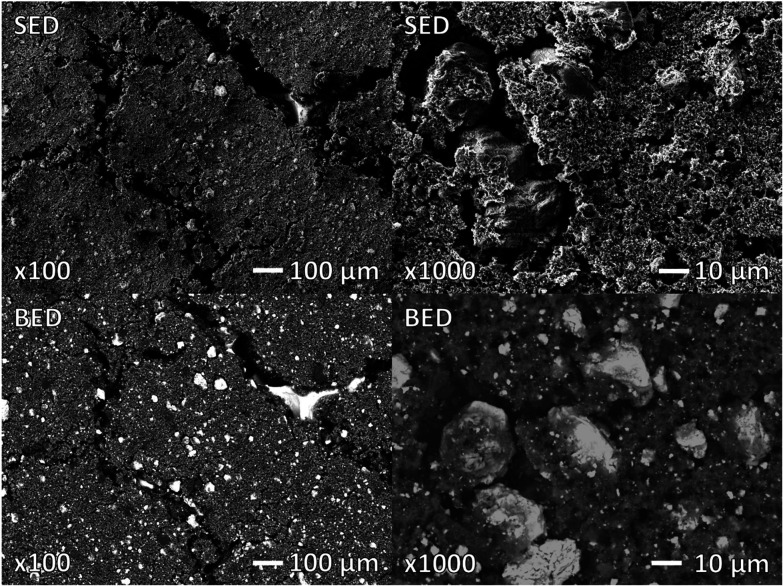
SEM after the application of 400 mA cm^−2^ during experiments in Fig. 11. This shows the limited effect of the experiments on the NiCu_0.2_ containing electrode in comparison with Fig. S1. Both secondary electron detector (SED) and backscatter electron detector (BED) modes of SEM are applied to show topography and composition, respectively.

Potential corrosion structural changes and the change in composition were determined using SEM and EDS. The used electrode (Fig. 13) displayed macroscale cracking in comparison with the unused electrode (Fig. S1). However, the Ni current collector remains largely covered, with limited loss of NiCu_0.2_ particles. The BED modus confirms the equivalent size and distribution of the NiCu_0.2_ material in the pristine and used electrodes. Additional particles were identified as KOH·2H_2_O and KHCO_3_ salts in accordance with XRD. EDS analysis reported a composition of NiCu_0.04_ for the electrode produced initially with NiCu_0.2_ particles. Therefore, under the alkaline oxidative conditions from the experiments, a significant amount of Cu dissolved. Nonetheless, the remaining Cu concentration suggests resistance to further corrosion. Such Cu dissolution and corrosion could result in copper complexes that are relatively stable, as indicated in eqn (9)–(11).^43,44^

9 Cu^2+^ + 2OH^−^ ⇄ CuO_(s)_ + H_2_O

10 3Cu^2+^ + 4OH^−^ ⇄ Cu_3_(OH)_4_^2+^

11 Cu^2+^ + 2NH_3_ + 2OH^−^ ⇄ Cu(NH_3_)_2_(OH)_2_

In a forthcoming publication, we will report on this NiCu_0.04_ stable composition resulting after prolonged oxidation in 6 M KOH and compare it with DFT calculations, indicating its stability against dissolution as shown in eqn (9)–(11). However, here we note that even in the presence of ammonia and nitrite, NiCu_0.04_ shows stable composition during prolonged experiments at industrial current densities.

### Dual product electrolyser

**Fig. 14:**
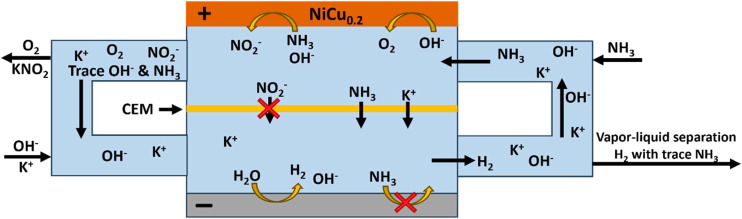
Schematic diagram of an AOR flow cell with a central liquid flow, resulting in cycling of K^+^ through the membrane. At the bottom negative electrode, water reduction takes place, producing H_2_ and OH^−^. Charge compensating K^+^ is transported through the CEM rather than protons under alkaline conditions. At the positive electrode, NH_3_ oxidation to NO_2_^−^ occurs using OH^−^ (and trace water oxidation to O_2_). The KOH and NH_3_ input flow channels provide feeds to enable the overall reaction KOH + H_2_O + NH_3_ → KNO_2_ + 3H_2_, as well as the (undesirable) OER. Ammonium could be transported back into the bottom cell, if the pH permits its presence (*e.g.*, at the outlet for KNO_2_ where OH^−^ becomes depleted).

This study demonstrated that it is possible to achieve high FE within 1 M NH_3_ with selective conversion of ammonia to NO_2_^−^. This significant reduction in potential in comparison with the OER presents a possible method for reducing the potential of an electrolyser, while making a value-added product, rather than the oxygen waste product. However, this requires ammonia in the catholyte, which makes product purification more complex. The setup can be further optimized by balancing the K^+^ transport through the CEM by flowing the catholyte into the anolyte (Fig. 14). The nitrite could further be used as a fertilizer, while the H_2_ could be used for energy storage or reinvested in the production of the consumed NH_3_.

Overall, this system could be interesting for small scale synthetic fertilizer production, especially if local electricity is produced in excess due to intermittence. The end product could be either KNO_2_ or NH_4_NO_2_, both of which are of value for contributing to the NPKS (nitrogen, phosphor, potassium, and sulphur) ratio of synthetic fertilizers. With KOH balanced within the system, the only input is ammonia, water, and KOH to compensate for product removal. The electrolyte could even be used directly if the pH is balanced through the addition of phosphoric acid and sulfuric acid. The addition of these acids would contribute to the NPKS ratio of the fertilizer. Farmers would then be able to produce their own specific ratio with limited transport costs and shorter supply chains. Furthermore, this would limit the storage and transport of ammonium nitrate, which is a potentially hazardous substance.

## Conclusions

Ni_0.8_Cu_0.2_(OH)_2_ has been investigated as a potential catalyst for ammonia oxidation to nitrite at industrially relevant current densities and concentrations. The Cu was homogeneously incorporated within the β-Ni(OH)_2_ phase to enhance the catalytic properties of Ni(OH)_2_. The prepared Ni_0.8_Cu_0.2_ has lower Tafel slopes of 49 and 140 mV dec^−1^ at lower and higher current densities, respectively, compared to 62 and 186 mV dec^−1^ for the undoped Ni(OH)_2_. In an undivided cell, the nitrite and nitrate yields are limited by the re-reduction of these products on the cathode. Furthermore, pH stabilisation with a supporting alkaline electrolyte is required to prevent pH reduction, which would also enable nitrite to nitrate oxidation.

In comparison, a Nafion® 117 cation exchange membrane divided cell under alkaline conditions results in consistent nitrite formation. This is only limited by ammonia depletion and the reduction of the pH to below 12. With this setup, the ammonia oxidation performance of NiCu_0.2_ significantly improved with respect to the yield and current densities. A faradaic efficiency towards nitrite of 67% at 2.5 mA cm^−2^ was achieved with 1 M ammonia in 6 M KOH. Furthermore, increasing the current density to 400 mA cm^−2^ improved the total faradaic efficiency to 96% with 95% NO_2_^−^ : NO_3_^−^ selectivity. This was achieved with 77% conversion in less than 3.5 hours. It was observed that the original Ni_0.83_Cu_0.17_ content was reduced in Cu content to Ni_0.96_Cu_0.04_ by the continuous ammonia oxidation. However, this composition is expected to remain stable, as it has also been observed in long term operation. The setup could be further optimised for synthetic fertilizer production, enabling small-scale fertilizer manufacture. To conclude, we have successfully developed a copper-doped nickel hydroxide material that achieves promising results under industrial relevant current density and NH_3_ concentration conditions. We suggest the application of a cation exchange membrane in future ammonia oxidation research to avoid skewing data towards N_2_ formation.

## Author contributions

Conceptualization: FM and DvN. Data acquisition and curation: DvN and PJ. Writing – original draft: DvN, PJ, and FM. Review and editing: DvN, PJ, AU, and FM. Funding acquisition: FM.

## Conflicts of interest

There are no conflicts to declare.

## Supplementary Material

GC-028-D5GC06877K-s001

## Data Availability

Data for this article are available at https://data.4tu.nl/datasets/eeb4617c-4c92-45d1-a0ab-57e41fb8889b/1.
