# Supplementary material for: Electrochemical ammonia oxidation using nickel copper hydroxide with H2 recovery at high current density and selectivity
Source: Green Chem. 2026 May 1;28(21):9013–25. doi: 10.1039/d5gc06877k (PMC13175027; doi:10.1039/d5gc06877k)
Supplement: GC-028-D5GC06877K-s001 [file GC-028-D5GC06877K-s001.pdf]

Supplementary Information (SI) for Green Chemistry.
This journal is © The Royal Society of Chemistry 2026

Supplementary Information

Electrochemical Ammonia Oxidation at Nickel Copper Hydroxide with H_2 Recovery at High Current Density and Selectivity

D.D. van Noordenne,[a] P.J. Jungbacker[a], A. Urakawa[a], and F.M. Mulder[a,*]

[a] Chemical Engineering, Faculty of Applied Sciences, Delft University of Technology, 2629 HZ Delft, the Netherlands

Supplementary note 1: Calculation of equilibrium potentials of relevant ammonia oxidation reactions

The calculation of equilibrium potentials with respect to the Reversible Hydrogen Electrode (RHE) in an alkaline electrolyte at pH 14 can be done by calculating the cell potentials E_{RHE} of a cell with the RHE as counter electrode. RHE itself at pH 14 has the half reaction:

$2H_2O + 2e^- \rightleftharpoons H_2 + 2OH^-$ E^0 = 0 V vs RHE @ pH 14 (0)

Equations (1)-(3) are given below again for convenience, while (4) is given for completion:

$NH_3(aq) + 3OH^- \rightleftharpoons ½N_2 + 3H_2O + 3e^-$ (1)

$NH_3(aq) + 7OH^- \rightleftharpoons NO_2^- + 5H_2O + 6e^-$ (2)

$NH_3(aq) + 9OH^- \rightleftharpoons NO_3^- + 6H_2O + 8e^-$ (3)

$N_2(g) + 12\ OH^- \rightleftharpoons 2NO_3^- + 6\ H_2O + 10\ e^-$ (4)

The potential difference with the RHE electrode can be determined by calculating ΔG and the cell potential for the two balanced equations (0) and

(1), (0) and (2), and (0) and (3), taking the number z of electrons involved into account.

The potential of (1) vs RHE can be determined from the difference between (0) and (1):

$2NH_3(aq) \rightleftharpoons N_2 + 3H_2$

ΔG = G(N_2) + G(H_2) – G(NH_3(Aq)) = 0 + 0 –(-26.57x2) = 53.1 kJ/mol

E_{RHE} = - ΔG/zF = -53.1e3/(6x96485)= -0.091 V with the negative sign stemming from the applied potential having the opposite direction of electron flow. The potential of the positive electrode where (1) runs is then +0.091V higher than where (0) runs. The potential of (1) at pH 14 versus SHE becomes:

(1) E_0 = -0.0592x14 –(-0.091) = -0.74 V vs SHE @ pH 14

The potential of (2) vs RHE can be determined from the difference between (0) and (2):

$NH_3(aq) + OH^- + H_2O \rightleftharpoons NO_2^- + 3\ H_2$

ΔG = G(NO_2^-(aq)) + G(3H_2) – (G(NH_3(aq))+G(OH^-) + G(H_2O))=

= -34.31 + 0 – (-26.57 – 157.32 – 237.2) = +386.8 kJ/mol

E_{RHE} = - ΔG/zF = -386.8e3 /(6x96485) = -0.67 V

The potential versus SHE, when (3) is at pH 14 then becomes:

(2) E_0= -0.0592x14 –(-0.67) = -0.16 V vs SHE @ pH 14

The potential of (3) vs RHE can be determined from the difference between (0) and (3):

$NH_3(aq) + OH^- + 2H_2O \rightleftharpoons NO_3^- + 4\ H_2$

ΔG = G(NO_3^-(aq)) + G(4H_2) – (G(NH_3(aq))+G(OH^-) + G(2H_2O))=

= -110.46 + 0 – (-26.57– 157.32 – 237.2x2) = 547.8 kJ/mol

E_{RHE} = - ΔG/zF = -547.8e3 /(8x96485) = -0.71 V

The potential versus SHE, when (3) is at pH 14 then becomes:

(3) E_0= -0.0592x14 –(-0.71) = -0.12 V vs SHE @ pH 14

The potential of (4) vs RHE can be determined from the difference between (0) and (4):

N_2(g) + 2 OH^- + 4 $H_2O \rightleftharpoons 2NO_3^-$ + $5\mathrm{H}_2$

ΔG = 2G(NO_3^-(aq)) + 5 G(H_2) – (G(N_2) + 2G(OH^-) + 4G($2H_2O$)) =

= -110.46x2 + 0 –(0 – 157.3x2 - 237.2x4) = 1042.48 kJ/mol

E_{RHE} = - ΔG/zF = -1042.48 e3 /(10x96485) = -1.08 V

The potential versus SHE, when (4) is at pH 14 then becomes:

(4) E_0= -0.0592x14 –(-1.08) = +0.25 V vs SHE @ pH 14

Although one may expect to observe (4) at similar potentials as the AOR reactions, in practice the direct di-nitrogen electrochemical oxidation has proven to be difficulties to be observed in any proposed catalytic system yet. For further information see [19].

Table S1. The performance of various electrocatalyst towards selective and high Faradaic efficient electrochemical ammonia oxidation. Underlined potential and current density are the applied values. Where applicable the FE is calculated from the reported product concentrations in the references and the (estimated average) current applied.

AOR Catalyst	Mem-brane	Coun-ter Electrode	Electrolyte	Ammonia	Potential (V)	Current Density (mA/cm²)	FE_{N2} (%)	$FE_{NO_2^-}$ (%)	$FE_{NO_3^-}$ (%)	Ref.
**IrO_2**	—	Ti	0.1 M Na_2SO_4, pH 7	0.5 M $(NH_4)_2SO_4$		<u>80</u>	4			[1]
**IrO_2**	Nafion® 424	Ti	0.1 M Na_2SO_4, pH 7	0.5 M $(NH_4)_2SO_4$		<u>80</u>	12			[1]
**IrO_2**	—	Ti	0.1 M Na_2SO_4, pH 7	1 M NH_4Cl		<u>80</u>	38			[1]
**IrO_2**	Nafion® 424	Ti	0.1 M Na_2SO_4, pH 7	1 M NH_4Cl		<u>80</u>	57			[1]
**IrO_2**	—	Ti	0.1 M Na_2SO_4, pH 12	0.5 M $(NH_4)_2SO_4$		<u>80</u>	35			[1]
**IrO_2**	Nafion® 424	Ti	0.1 M Na_2SO_4, pH 12	0.5 M $(NH_4)_2SO_4$		<u>80</u>	67			[1]
**IrO_2**	—	Ti	0.1 M Na_2SO_4, pH 12	1 M NH_4Cl		<u>80</u>	42			[1]
**IrO_2**	Nafion® 424	Ti	0.1 M Na_2SO_4, pH 12	1 M NH_4Cl		<u>80</u>	85			[1]
**Pt-Ir-Rh**	—	Pt-Ir-Rh	0.2 M KOH	21.5 mM NH_4OH	<1 Cell	<u>25</u>	92			[2]
**NiCu**	—	Pt/C	1 M NaOH, pH 12	29 mM NH_4Cl	<u>1.0 Cell</u>		93			[3]
**NiCu**	—	Pt/C	1 M NaOH, pH 12	29 mM NH_4Cl	<u>1.1 Cell</u>		81			[3]
**Ni foam**	—	IrO_2/Ti plate	0.1 M Na_2SO_4, pH 11	0.6 mM $(NH_4)_2SO_4$	<u>0.8 vs. Hg/HgO</u>		3	0	19	[4]
**NiCuFeOOH**	—	Pt foil	0.5 NaOH, pH 13.6	55 mM NH_4Cl	<u>0.4 vs. SCE</u>		41			[5]
**NiCuFeOOH**	—	Pt foil	0.5 NaOH,	55 mM NH_4Cl	<u>0.55 vs. SCE</u>		15			[5]

			pH 13.6							
**Cu**	Glass frit	Pt wire	0.01 M KOH	0.1 M NH_4OH	1.8 vs. RHE	2		85	0	[6]
**Cu**	Glass frit	Pt wire	0.01 M KOH	0.1 M NH_4OH	1.7 vs. RHE	5-40		9	44	[6]
**Cu**	Glass frit	Pt wire	0.11 M KOH	0.1 M NH_4OH	1.7 vs. RHE	2		86	0	[6]
**Cu**	Glass frit	Pt wire	0.11 M KOH	0.1 M NH_4OH	1.8 vs. RHE	4		62	5	[6]
**Cu**	Glass frit	Pt wire	0.11 M KOH	0.1 M NH_4OH	2.0 vs. RHE	25-40		36	29	[6]
**Cu**	Glass frit	Pt wire	1.1 M KOH	0.1 M NH_4OH	1.7 vs. RHE			55	0	[6]
**Cu**	Glass frit	Pt wire	1.1 M KOH	0.1 M NH_4OH	1.8 vs. RHE	4		27	9	[6]
**V_o-rich CuO[a]**	—	Carbon rod	1 M KOH	1 M NH_4OH	0.6 vs. Hg/HgO		63	34	0	[7]
**V_0-Poor CuO[a]**	—	Carbon rod	1 M KOH	1 M NH_4OH	0.6 vs. Hg/HgO		26	72	0	[7]
**Pt**	—	Ti plate	0.1 M Na_2SO_4	1.5 mM $(NH_4)_2SO_4$		1	5	0	0	[8]
**Ni**	—	Ti plate	0.1 M Na_2SO_4	1.5 mM $(NH_4)_2SO_4$		1	4	8	50	[8]
**Cu**	—	Ti plate	0.1 M Na_2SO_4	1.5 mM $(NH_4)_2SO_4$		1	6	0	54	[8]
**PtNi**	—	Ti plate	0.1 M Na_2SO_4	1.5 mM $(NH_4)_2SO_4$		1	5	13	25	[8]
**PtCu**	—	Ti plate	0.1 M Na_2SO_4	1.5 mM $(NH_4)_2SO_4$		1	31	1	38	[8]
**PtCuNi**	—	Ti plate	0.1 M Na_2SO_4	1.5 mM $(NH_4)_2SO_4$		1	5	17	22	[8]
**PtCu**	—	Ti plate	0.1 M Na_2SO_4	1.5 mM $(NH_4)_2SO_4$		2	1	7	44	[8]
**PtCu**	—	Ti plate	0.1 M Na_2SO_4	5 mM $(NH_4)_2S$		1	5	28	48	[8]

				O_4						
**Cu**	P4 Ceramic frit	Pt/Ti mesh	0.1 M KOH	0.1 M NH_4OH	2.0 vs. RHE			0	18	[9]
**Ni**	P4 Ceramic frit	Pt/Ti mesh	0.1 M KOH	0.1 M NH_4OH	2.0 vs. RHE			8	19	[9]
**$Ni(OH)_2$**	Fumasep FAB-PK-130	Ni foam	0.1 M Na_2SO_4	0.2 M NH_4OH	1.6 vs. RHE	6	53	28	18	[10]
**$Ni(OH)_2$**	Fumasep FAB-PK-130	Ni foam	0.1 M Na_2SO_4	0.2 M NH_4OH	1.9 vs. RHE	18	89	9	42	[10]
**$Ni(OH)_2$**	Fumasep FAB-PK-130	Ni foam	0.1 M Na_2SO_4, pH 12.8	0.2 M NH_4OH	1.9 vs. RHE			39	9	[10]
**$Ni(OH)_2$**	Fumasep FAB-PK-130	Ni foam	0.1 M NaOH	0.2 M NH_4OH	1.6 vs. RHE	10	34	58	1	[10]
**$Ni(OH)_2$**	Fumasep FAB-PK-130	Ni foam	0.1 M NaOH	0.2 M NH_4OH	1.9 vs. RHE	28	20	39	9	[10]
**SnO_2**	—	Pt Foil	0.5 M K_2SO_4, pH 11	5 mM $(NH_4)_2SO_4$	1.45 vs. RHE	1	84	7	3	[11]
**SnO_2**	—	Pt Foil	0.5 M K_2SO_4, pH 11	5 mM $(NH_4)_2SO_4$	1.75 vs. RHE	1	25	4	16	[11]
**$Ni(OH)_2/SnO_2$**	—	Pt Foil	0.5 M K_2SO_4, pH 11	5 mM $(NH_4)_2SO_4$	1.45 vs. RHE	1	90	1	0	[11]
**$Ni(OH)_2/SnO_2$**	—	Pt Foil	0.5 M K_2SO_4, pH 11	5 mM $(NH_4)_2SO_4$	1.75 vs. RHE	2	43	0	31	[11]
**$Ni(OH)_2$**	—	Pt Foil	0.5 M K_2SO_4, pH 11	5 mM $(NH_4)_2SO_4$	1.45 vs. RHE	1	93	9	1	[11]
**$Ni(OH)_2$**	—	Pt Foil	0.5 M K_2SO_4, pH 11	5 mM $(NH_4)_2SO_4$	1.75 vs. RHE	1	24	4	1	[11]
**Ni**	P4	Pt/Ti	0.1 M	0.5 M	1.0 vs.			14	5	[12]

	glass frit	Mesh	K_2HPO_4	NH_4OH	SHE					
**β-NiOOH**	P4 glass frit	Pt/Ti Mesh	0.1 M K_2HPO_4	0.5 M NH_4OH	1.0 vs. SHE			13	16	[12]
**CoO_xH_y/ β-NiOOH**	P4 glass frit	Pt/Ti Mesh	0.1 M K_2HPO_4	0.5 M NH_4OH	1.0 vs. SHE	2		38	41	[12]
**Cu**	—	Pt foil	0.5 M K_2SO_4, pH 11	5 mM $(NH_4)_2SO_4$	1.65 vs. RHE		42	2	49	[13]
**Cu_2Co_1**	—	Pt foil	0.5 M K_2SO_4, pH 11	5 mM $(NH_4)_2SO_4$	1.65 vs. RHE		73	1	26	[13]
**Cu_1Co_4**	—	Pt foil	0.5 M K_2SO_4, pH 11	5 mM $(NH_4)_2SO_4$	1.65 vs. RHE		60	0	40	[13]
**Co**	—	Pt foil	0.5 M K_2SO_4, pH 11	5 mM $(NH_4)_2SO_4$	1.55 vs. RHE		65	3	32	[13]
**CuCo**	—	Pt foil	0.5 M K_2SO_4, pH 11	5 mM $(NH_4)_2SO_4$	1.65 vs. RHE		77	1	23	[13]
**Ni**	—	IrO_2	0.1 M Na_2SO_4	1.5 mM $(NH_4)_2SO_4$		2	3	2	8	[14]
**$Ni_{400\,°C}$[b]**	—	IrO_2	0.1 M Na_2SO_4	1.5 mM $(NH_4)_2SO_4$		2	10	4	22	[14]
**Ag/$Ni_{400\,°C}$[b]**	—	IrO_2	0.1 M Na_2SO_4	1.5 mM $(NH_4)_2SO_4$	1.8V vs. RHE	2	32	5	19	[14]
**Ni foam**	—	DSA[c]	10 mM Na_2SO_4, pH 11	2 mM $(NH_4)_2SO_4$	0.7 vs. Hg/HgO		5	64	14	[15]
**Ni foam**	—	DSA[c]	10 mM Na_2SO_4, pH 11	2 mM $(NH_4)_2SO_4$	1.0 vs. Hg/HgO		1	3	47	[15]
**Cu/NF**	—	DSA[c]	10 mM Na_2SO_4, pH 11	2 mM $(NH_4)_2SO_4$	0.7 vs. Hg/HgO		15	59	28	[15]
**Cu/NF**	—	DSA[c]	10 mM Na_2SO_4, pH 11	2 mM $(NH_4)_2SO_4$	1.0 vs. Hg/HgO		5	2	81	[15]
**$Ni(OH)_2$-$Cu(OH)_2$ / C**	—	Pt	0.1 M NaOH	50 mM NH_4Cl	0.55 vs. Ag/AgCl	9-2		0	9	[16]

**Cu_8Ni_2**	—	Pt	0.5 M KOH	55 mM NH_4Cl	<u>1.6V vs RHE</u>	53		67		[17]
**CuNi-‘L@(−196)’**	—	Pt	0.5 M KOH	55 mM NH_4Cl	<u>1.7V vs RHE</u>	106-28		5		[18]

[a] Oxygen vacancies; [b] Annealed at 400 °C; [c] Dimensionally stable anode

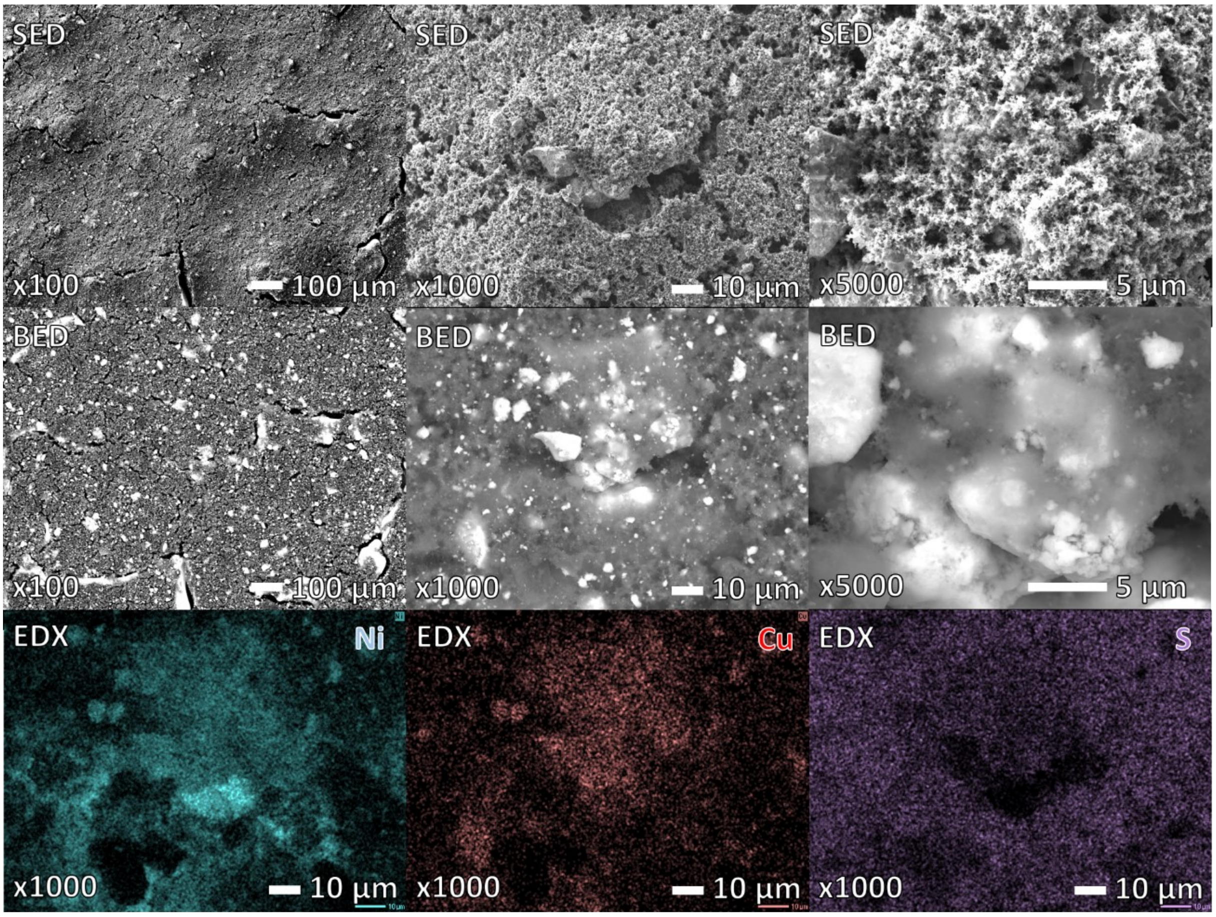

Figure S1. EDS and SEM of an unused $NiCu_{0.2}$ electrode, showing the particle distribution within the electrode. High porosity is achieved through the phase inversion method. Reduction in K_2SO_4 contamination is visualized with EDS of S. Both secondary electron detector (SED) and backscatter electron detector (BED) modes of SEM are applied to show topography and composition, respectively.

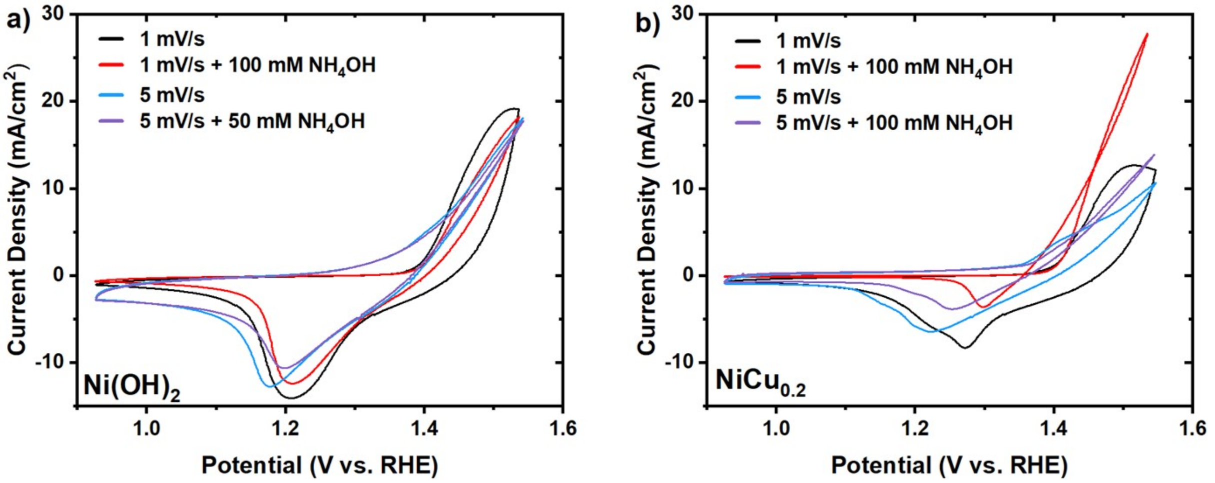

Figure S2. Cyclic voltammetry of a) $Ni(OH)_2$ and b) $NiCu_{0.2}$ in 1 M KOH with and without NH_4OH at both 1 mV/s and 5 mV/s Scan rate.

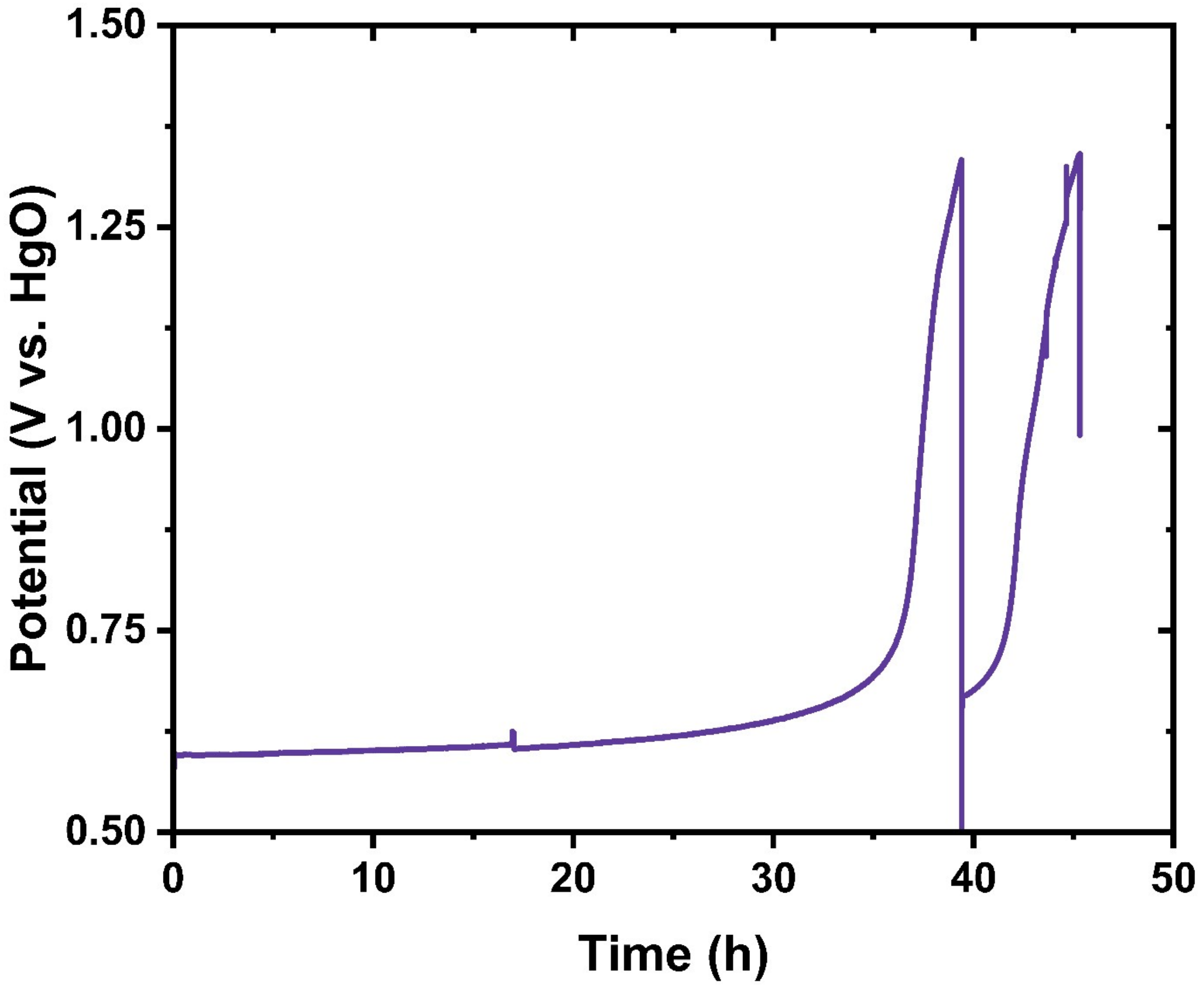

Figure S3. chronopotentiometry at 10 mA/cm^2 in 1 M KOH divided by a nafion 117 membrane. when the potential peaked, the cell was shut-off. at that moment Ir was 1000 ohm and pH of 8. Cell recovers slowly over time due to concentration gradient resulting in transport back. Cell was turned on again after 24 hours and spliced after the initial shutdown.

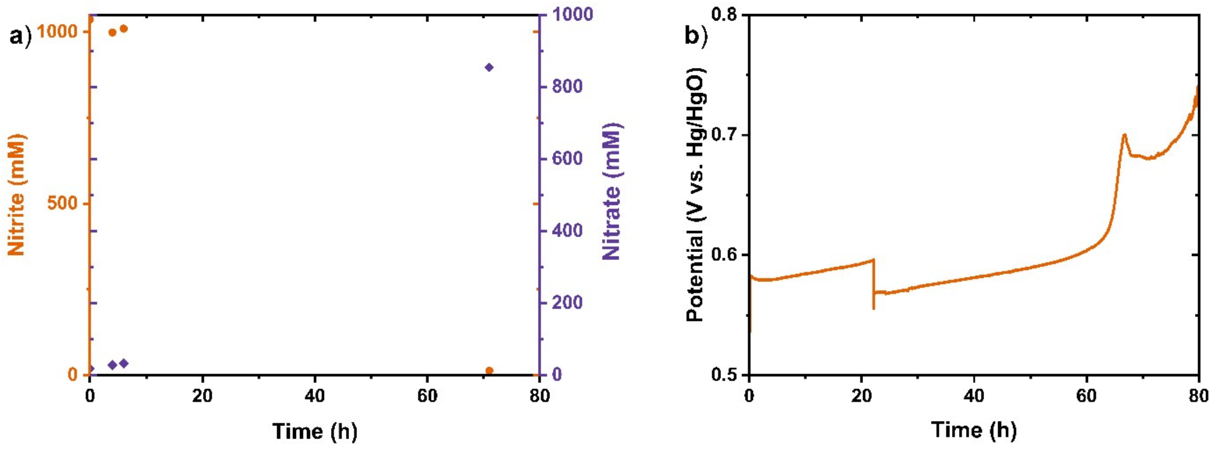

Figure S4. a) Nitrite and nitrate concentration related to chronopotentiometry in 1M KOH with 1 M KNO_2 at 10 mA/cm^2. b) Potential measured during the chronopotentiometric measurement in 2x50 mL Nafion 117 membrane cell.

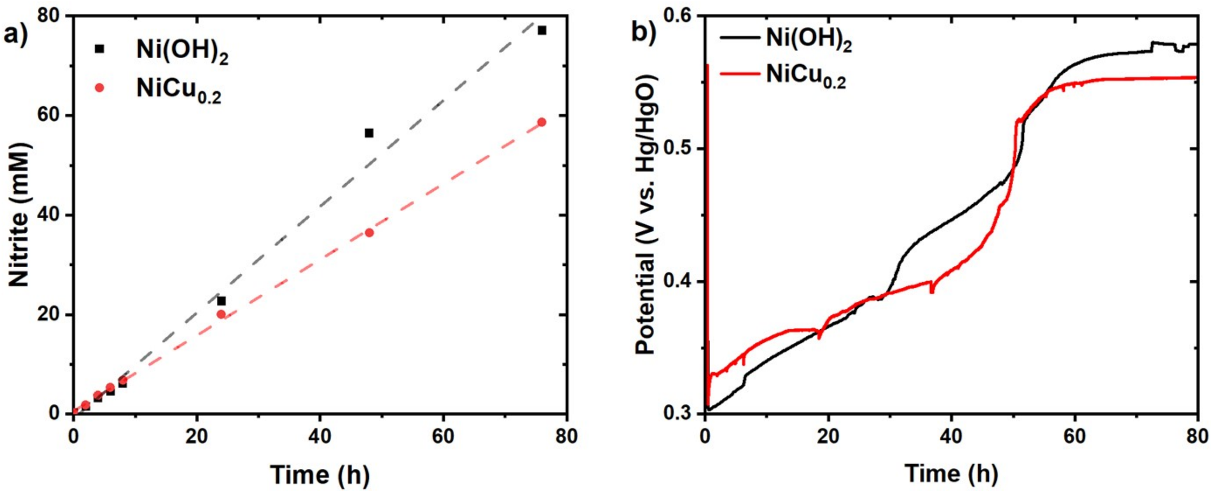

Figure S5. Chronopotentiometry of $Ni(OH)_2$ and $NiCu_{0.2}$ in 6 M KOH with 1 M NH_3 at 2.5 mA/cm^{2}with 2x50 mL 117 Nafion cell. a) measured nitrite concentration and b) potential during the chronopotentiometry. Showing the increased production rate on $Ni(OH)_2$ due to the increased potential.

References

[1] K.-W. Kim, Y.-J. Kim, I.-T. Kim, G.-I. Park, E.-H. Lee, "Electrochemical conversion characteristics of ammonia to nitrogen" *Water Research* **2006**, *40*, 1431–1441.

[2] E. P. Bonnin, E. J. Biddinger, G. G. Botte, "Effect of catalyst on electrolysis of ammonia effluents" *Journal of Power Sources* **2008**, *182*, 284–290.

[3] W. Xu, D. Du, R. Lan, J. Humphreys, D. N. Miller, M. Walker, Z. Wu, J. T. S. Irvine, S. Tao, "Electrodeposited NiCu bimetal on carbon paper as stable non-noble anode for efficient electrooxidation of ammonia" *Applied Catalysis B: Environmental* **2018**, *237*, 1101–1109.

[4] Y.-J. Shih, Y.-H. Huang, C. P. Huang, "Electrocatalytic ammonia oxidation over a nickel foam electrode: Role of Ni(OH)2(s)-NiOOH(s) nanocatalysts" *Electrochimica Acta* **2018**, *263*, 261–271.

[5] M. Zhu, Y. Yang, S. Xi, C. Diao, Z. Yu, W. S. V. Lee, J. Xue, "Deciphering NH3 Adsorption Kinetics in Ternary Ni–Cu–Fe Oxyhydroxide toward Efficient Ammonia Oxidation Reaction" *Small* **2021**, *17*, 2005616.

[6] S. Johnston, L. Kemp, B. Turay, A. N. Simonov, B. H. R. Suryanto, D. R. MacFarlane, "Copper-Catalyzed Electrosynthesis of Nitrite and Nitrate from Ammonia: Tuning the Selectivity via an Interplay Between Homogeneous and Heterogeneous Catalysis" *ChemSusChem* **2021**, *14*, 4793–4801.

[7] J. Huang, Z. Chen, J. Cai, Y. Jin, T. Wang, J. Wang, "Activating copper oxide for stable electrocatalytic ammonia oxidation reaction via in-situ introducing oxygen vacancies" *Nano Res.* **2022**, *15*, 5987–5994.

[8] S. S. Prabowo Rahardjo, Y.-J. Shih, "Electrocatalytic Ammonia Oxidation Mediated by Nickel and Copper Crystallites Decorated with Platinum Nanoparticle (PtM/G, M = Cu, Ni)" *ACS Sustainable Chem. Eng.* **2022**, *10*, 5043–5054.

[9] S. Johnston, S. Cohen, C. K. Nguyen, K. N. Dinh, T. D. Nguyen, S. Giddey, C. Munnings, A. N. Simonov, D. R. MacFarlane, "A Survey of Catalytic Materials for Ammonia Electrooxidation to Nitrite and Nitrate" *ChemSusChem* **2022**, *15*, e202200614.

[10] J. J. Medvedev, Y. Tobolovskaya, X. V. Medvedeva, S. W. Tatarchuk, F. Li, A. Klinkova, "Pathways of ammonia electrooxidation on nickel hydroxide anodes and an alternative route towards recycled fertilizers" *Green Chem.* **2022**, *24*, 1578–1589.

[11] J. Hou, Y. Cheng, H. Pan, P. Kang, "Tailored Bimetallic Ni–Sn Catalyst for Electrochemical Ammonia Oxidation to Dinitrogen with High Selectivity" *Inorg. Chem.* **2023**, *62*, 3986–3992.

[12] S. Cohen, S. Johnston, C. K. Nguyen, T. D. Nguyen, D. A. Hoogeveen, D. V. Zeil, S. Giddey, A. N. Simonov, D. R. MacFarlane, "A CoOxHy/β-NiOOH electrocatalyst for robust ammonia oxidation to nitrite and nitrate" *Green Chem.* **2023**, *25*, 7157–7165.

[13] J. Cui, J. Hou, H. Pan, P. Kang, "Self-supporting CuM LDH (M = Ni, Fe, Co) carbon cloth electrodes for selective electrochemical ammonia oxidation to nitrogen" *Journal of Electroanalytical Chemistry* **2023**, *940*, 117502.

[14] S. S. P. Rahardjo, Y.-J. Shih, "Electrochemical characteristics of silver/nickel oxide (Ag/Ni) for direct ammonia oxidation and nitrogen selectivity in paired electrode system" *Chemical Engineering Journal* **2023**, *452*, 139370.

[15] M.-H. Tsai, Y. Juang, C.-C. Hu, L.-C. Hua, B. K. Mahata, C. Huang, "The direct electrocatalytic oxidation of ammonia by copper-deposited nickel foam catalysts" *Electrochimica Acta* **2023**, *446*, 142130.

[16] W. Xu, R. Lan, D. Du, J. Humphreys, M. Walker, Z. Wu, H. Wang, S. Tao, "Directly growing hierarchical nickel-copper hydroxide nanowires on carbon fibre cloth for efficient electrooxidation of ammonia" *Applied Catalysis B: Environmental* **2017**, *218*, 470–479.

[17] Z. Mao, Y. Tian, B. Guo, R. Chen, Y. Zeng, F. Hou, X. Yan, J. Liang, "Modulation of charge distribution enabling CuNi nano-alloys for efficient ammonia oxidation reaction to nitrite production" *Chemical Engineering Journal* **2024**, *484*, 149570.

[18] Y. Tian, R. Chen, X. Liu, Z. Mao, H. Tan, D. Yang, F. Hou, X. Liu, L. Yin, X. Yan, J. Liang, "Highly stabilized and selective ammonia electro-oxidation over CuNi metallic glass nanoarray" *Carbon Energy* **2025**, *7*, e667.

[19] P. Jungbacker, R. Kortlever and F. M. Mulder, *Green Chem.*, **2026**, DOI: 10.1039/D5GC05480J
